# Modeling selectivity of antimicrobial peptides: how it depends on the presence of host cells and cell density

**DOI:** 10.1039/d3ra06030f

**Published:** 2023-11-22

**Authors:** Suemin Lee, Bethany R. Schefter, Sattar Taheri-Araghi, Bae-Yeun Ha

**Affiliations:** a Department of Physics and Astronomy, University of Waterloo Waterloo Ontario N2L 3G1 Canada byha@uwaterloo.ca; b Department of Physics and Astronomy, University of Western Ontario London Ontario N6A 3K7 Canada; c Department of Physics and Astronomy, California State University Northridge CA 91330 USA

## Abstract

Antimicrobial peptides (AMPs), naturally-occurring peptide antibiotics, are known to attack bacteria selectively over the host cells. The emergence of drug-resistant bacteria has spurred much effort in utilizing optimized (more selective) AMPs as new peptide antibiotics. Cell selectivity of these peptides depends on various factors or parameters such as their binding affinity for cell membranes, peptide trapping in cells, peptide coverages on cell membranes required for membrane rupture, and cell densities. In this work, using a biophysical model of peptide selectivity, we show this dependence quantitatively especially for a mixture of bacteria and host cells. The model suggests a rather nontrivial dependence of the selectivity on the presence of host cells, cell density, and peptide trapping. In a typical biological setting, peptide trapping works in favor of host cells; the selectivity increases with increasing host-cell density but decreases with bacterial cell density. Because of the cell-density dependence of peptide activity, the selectivity can be overestimated by two or three orders of magnitude. The model also clarifies how the cell selectivity of AMPs differs from their membrane selectivity.

## Abstract 2

Antimicrobial peptides (AMPs), naturally-occurring peptide antibiotics, are known to attack bacteria selectively over the host cells.

## Graphical abstract

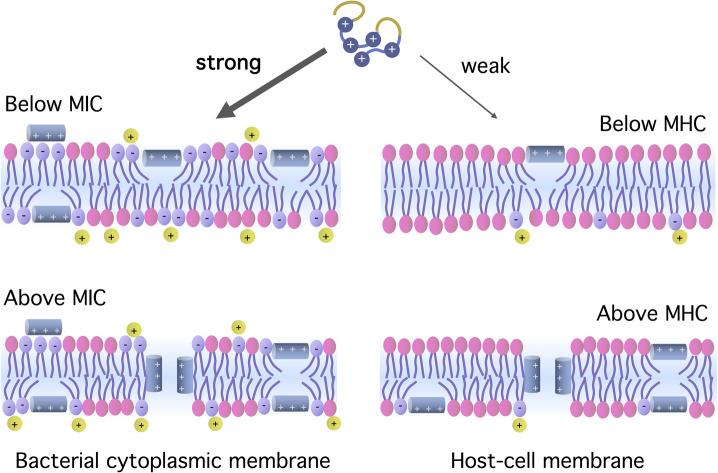

## 1. Introduction

**Fig. 1:**
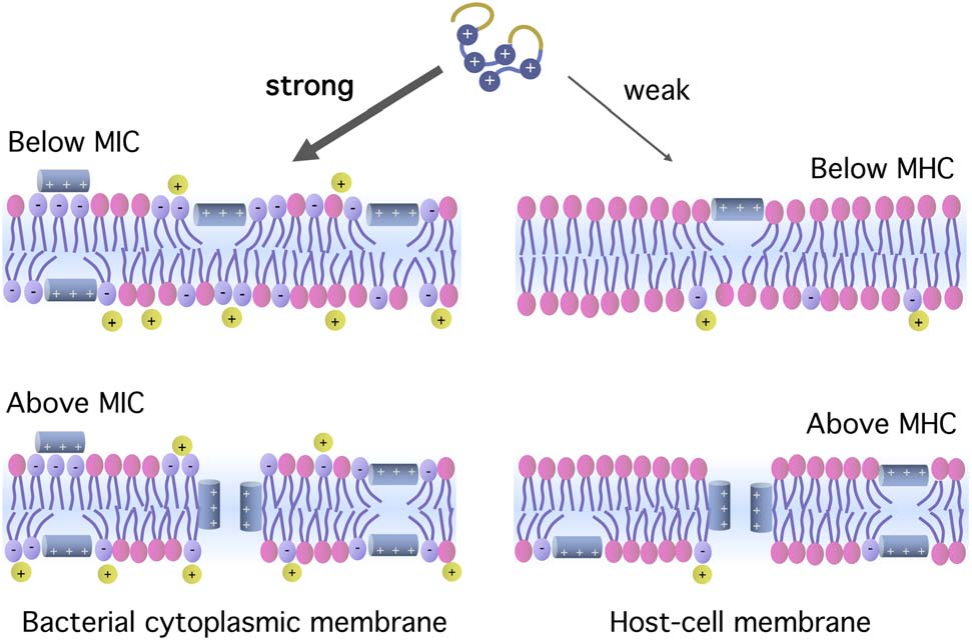
Origin of peptide selectivity. Cationic antimicrobial peptides interact more strongly with bacterial membranes enriched with anionic lipids. Bound peptides can form pores in the membrane when the bulk concentration is at or above the MIC or MHC. In the figure, the membranes are only schematically illustrated, leaving out such details as membrane proteins and the presence of cholesterol in the host-cell membrane. The figure is inspired by ref. 1, 2 and 10.

Antimicrobial peptides (AMPs) are naturally-occurring peptide antibiotics used in the host defense of living organisms (*e.g.*, animals, plants, …).^1,2^ They are relatively short, typically consisting of 20–50 amino acids. In the bulk, they often resemble random coils, but when inserted in membranes, they assume compact, amphiphilic structures (*e.g.*, *α* helices), as required for their antimicrobial activity (*e.g.*, membrane perturbation). AMPs are mostly cationic and thus utilize the unique ‘design feature’ of microbial membranes,^1^ enriched with anionic lipids.^1–3^ Cationic AMPs preferentially attach to and rupture microbial membranes over host cell membranes; in the latter case, anionic lipids are segregated to their inner layer (see Fig. 1). Once they gain entry into the cytoplasm, they can target key intra-cellular components (*e.g.*, DNA and proteins), leading to intra-cellular killing of microbes.^1,2^

There has been much interest in developing enhanced AMPs as potent peptide antibiotics, especially for fighting drug-resistant bacteria.^1–5^ Membrane-targeting AMPs are advantageous.^1–4,6^ They act *via* physical mechanisms such as pore formation^1–4,6^ or anionic-lipid clustering^3^ in membranes, which bacteria cannot easily avoid. In addition to rupturing bacterial membranes, they act as metabolic inhibitors^1,2^ and/or immunomodulators.^7^ Even though pathogens can, in principle, evolve antimicrobial resistance,^8,9^ the therapeutic potential of these multitasking molecules deserves much consideration.^4,5^

Cationic AMPs can single out bacteria through their stronger binding affinity for bacterial membranes.^1–4,6^ The resulting selectivity can be quantified by the ratio of two concentrations: the minimum hemolytic concentration (MHC) and the minimum inhibitory concentration (MIC).^10,11^ At or beyond this concentration, peptides can form pores in their binding membranes, as illustrated in Fig. 1. The larger the ratio MHC/MIC is for a given peptide, the more selective the peptide is. In a sizeable range of peptide concentration (∼μM) between MIC and MHC, the peptide is active against bacteria while leaving the host cells unharmed.

The selectivity of AMPs is influenced by a number of factors or parameters such as their binding affinity for cell membranes, peptide trapping in (dead) cells,^12–14^ cell density,^11–18^ and a peptide coverage on cell membranes required for membrane rupture.^19–21^ Let *P*/*L* denote the molar ratio of bound peptides to lipids. At the MIC or MHC, *P*/*L* reaches a threshold value, *P*/L*. The value of *P*/L* depends on the type of peptide and lipid^19–21^ and is typically larger for membranes containing lipids with smaller headgroups such as phosphatidylethanolamine (PE) as in bacterial membranes. Recent studies suggest that at *P*/L*, each cell consumes a certain number of peptides with some of them trapped in the cell.^12–14^ This implies that the MIC or the MHC increases with increasing cell density; as a result, the ratio MHC/MIC is cell-density dependent.^11–13,16–18^ The cell-density dependence is often referred to as an inoculum effect^12–15^ and is known to enhance population survivability.^14^

A natural consequence of the cell-density dependence of peptide activity and selectivity is that the selectivity depends on the way it is measured.^16–18^ For instance, it can be obtained by combining MIC and MHC measured separately from bacteria-only and host-cell-only solutions, respectively. In this work, the resulting selectivity is referred to as “noncompetitive” selectivity. More realistically, it can be measured from a mixture of both types of cells: “competitive” selectivity. In this case, the presence of host cells raises the MIC and influences the ratio MHC/MIC.^13,16,22^ These two approaches generally lead to different levels of selectivity. This implies that the selectivity reflects the biological setting of infected sites (*e.g.*, the degree of infection, …).

According to what is discussed above, peptide selectivity not only reflects peptide's intrinsic properties such as peptide charge and hydrophobicity, but it also depends on external parameters such as cell density and the presence of host cells. Does this mean that the selectivity should be measured for a wide range of cell density and various combinations of host cell and bacterial cell density? Recent modeling efforts, however, suggest that these two aspects (intrinsic and extrinsic) are well separated.^16,18^ With an appropriate model, one can figure out the selectivity with varying cell density, once it is known at a low cell-density limit or at conveniently-chosen density. Furthermore, in the past, model lipid membranes, mimicking cell membranes, were often used for peptide activity or selectivity experiments.^10,19–21^ How does the resulting membrane selectivity differ from cell selectivity measured for cells (bacteria *versus* host cells)? Peptide trapping is one of the determining factors in the latter^12–14,16^ but is expected to be insignificant in the former.

Recently, we examined theoretically peptide selectivity and clarified the effects of peptide trapping on the selectivity, MHC/MIC.^16^ This effort is relevant in the presence of an excess amount of host cells or for a homogeneous solution of either bacteria or host cells. Here, we extend this effort and offer a more complete picture of the activity and selectivity of AMPs, which can be used to interpret selectivity measurements or to assist with our endeavor in finding optimized peptides.

This work builds on earlier studies.^12–16^ The results reported in this work, which are relevant for melittin-like peptides, suggest a rather nontrivial dependence of the selectivity on the presence of host cells, peptide trapping, and cell density. Peptide trapping can enhance or reduce the selectivity depending on how cell (host and bacterial) density is chosen. In most cases, it works in favor of the host cells, enhancing the selectivity. The presence of an excess amount of host cells (5 × 10^9^ cells per mL) as in whole blood can raise the MIC more than 10-fold, proportionally with the density of bacterial cells. The resulting MIC still falls in a low-μM range as long as the bacterial cell density is somewhat smaller than 5 × 10^7^ cells per mL.

Let *C*_B_ and *C*_H_ be the density of bacteria and the density of host cells, respectively, and *N*_p_ the number of peptides trapped per cell. As we raise *C*_B_ and *C*_H_ coherently so that *C*_B_ = *C*_H_, the selectivity decreases in both noncompetitive and competitive cases. Similarly, in the presence of an excess amount of host cells, the selectivity decreases with increasing *C*_B_ in both cases, more so for larger *N*_p_. In contrast, when the bacterial cell density is fixed at *C*_B_ = 5 × 10^4^ cells per mL or *C*_B_ = 10^8^ cells per mL, the selectivity increases with increasing *C*_H_, more rapidly for larger *N*_p_. Compared to the competitive one, the noncompetitive selectivity can be overestimated by more than two orders of magnitude, depending on how *C*_B_ and *C*_H_ are chosen (see refs. 11 and 16 for related discussions).

We also clarify how the cell selectivity of AMPs differs from their membrane selectivity. While the selectivity based on model membranes is typically larger than the corresponding cell selectivity, the (relative) difference between competitive and noncompetitive selectivity is generally larger in the latter. Except for some differences, membrane selectivity and cell selectivity of AMPs are qualitatively similar to each other. If interpreted with care, the former can provide useful information about the latter.

In this work, we will focus our effort on presenting a selectivity model in a pedagogical but yet systematic manner. In our consideration, one of the main differences between model membranes and cells comes from peptide trapping in the latter. Nevertheless, we will use membrane density and cell density interchangeably; also MICs and MHCs refer to peptide concentration beyond which membranes are ruptured, whether they are model membranes or cell membranes.

This paper is organized as follows: in Section 2, we present a simple picture of how the activity and selectivity of AMPs vary with cell density for a noncompetitive and competitive medium. Section 3 introduces a Langmuir model of peptide binding. Section 4 summaries the results for peptide activity and selectivity as a function of cell density; the effect of peptide trapping is highlighted, and membrane selectivity and cell selectivity are compared. All the symbols and acronyms are defined in Table 1.

**Table 1:** Definitions of symbols and acronyms

Symbol or acronym	Definition
*A*_cell_	Cell surface area
*A*_B_ (*A*_H_)	Bacterial (host) cell surface area
*a*_l_	Lipid headgroup area
*a*_B_ (*a*_H_)	Lipid headgroup area of bacterial (host-cell) membranes
*A*_p_	Cross-sectional area of a peptide on the membrane surface
*C*_cell_	Concentration of cells
*C*_H_ (*C*_B_)	Concentration of host (bacterial) cells
*C*_p_	Total peptide concentration
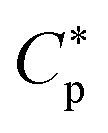	*C*_p_ at which *P*/*L* = *P*/L*
*P*/*L*	Molar ratio of bound peptides to lipids
*P*/L*	Threshold value of *P*/*L* required for membrane rupture, corresponding to MIC or MHC
MIC	Minimum inhibitory concentration
MHC	Minimum hemolytic concentration
MIC_0_ (MHC_0_)	MIC (or MHC) in the low-cell density limit: *C*_cell_ → 0
*μ*_bound_	Chemical potential of bound peptides
*μ*_free_	Chemical potential of free peptides
*N*_p_	Number of trapped peptides per cell
*N*_pB_ (*N*_pH_)	Number of trapped peptides per bacterial (host) cell
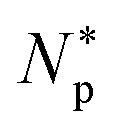	Number of trapped peptides per cell at *P*/L*
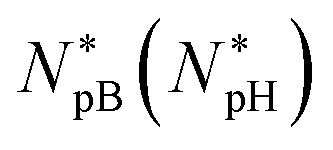	Number of trapped peptides per bacterial (host) cell at *P*/L*
*n*_p_	Number density of trapped peptides in a cell
*n*_pB_ (*n*_pB_)	Number density of trapped peptides in a bacterial (host) cell
*N*_cell_	Number of cells
*σ*_p_	Planar density of adsorbed peptides to membranes
*k*_B_	Boltzmann constant
*T*	Temperature
*V*	Volume of the system
*v*_p_	Volume of each peptide
*V*_cell_	Volume of each cell
*w*	Binding energy of a peptide on membranes
*w*_B_ (*w*_H_)	Binding energy of a peptide on bacterial (host) cell membranes
*u*	Trapping energy of a peptide in a cell
*u*_B_ (*u*_H_)	Trapping energy of a peptide in a bacterial (host) cell

## 2. Cell and membrane selectivity of antimicrobial peptides

In this section, we present a pedagogical approach to peptide activity and selectivity, which shows how peptide selectivity depends on cell density and peptide trapping in cells. We start with a homogeneous system of either bacterial or host cells, referred to as a noncompetitive case, and turn to a mixture of both types of cells, referred to as a competitive case.

Before proceeding further, we introduce several parameters relevant for peptide activity and selectivity. A key “extrinsic” parameter is the number density of peptides, denoted as *C*_p_; so is the density of cells, *C*_cell_.^12,14,17,18^ The surface area of each cell, *A*_cell_, matters.^17^ In terms of the number of membrane-bound peptides, doubling *A*_cell_ for given *C*_cell_ is equivalent to doubling *C*_cell_ for given *A*_cell_. The peptide selectivity arises primarily from the difference in binding energy, denoted as *w*, between bacterial membranes and host-cell membranes.^1,16–18^ Membrane rupture occurs in an all-or-none *C*_cell_-dependent manner.^19,20,23^ Recall that *P*/*L* is the molar ratio of membrane-bound peptides to lipids. At a certain value of *C*_p_, *i.e.*, 
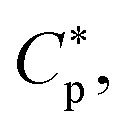
*P*/*L* reaches a threshold value required for membrane rupture, *P*/L*;^10,19–21^
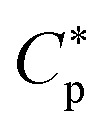
 is either MIC or MHC. Finally, *N*_p_ denotes the number of trapped peptides per cell. This needs to be taken with caution. Below 
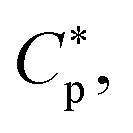
 we assume that *N*_p_ = 0. In this case, penetration of peptides into a cell is expected to be a rare event, since it involves overcoming a large free energy barrier for crossing an otherwise intact cell membrane. At 
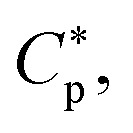
 half of the cell membranes are ruptured. Thus, *N*_p_ can be interpreted as the number of peptides trapped in each dead cell. Alternatively, it can be considered as the “average” number of peptides trapped per cell at 
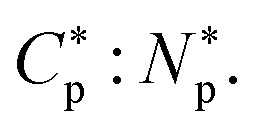
 Here, we employ this definition of *N*_p_, which is half of the number of trapped peptides in a dead cell. Beyond, *N*_p_ can be larger than 
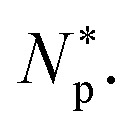
 But we ignore the possible weak dependence of *N*_p_ on *C*_p_ (see Section 2.2 for further discussion). As a result, for 
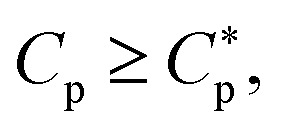
 we use *N*_p_ and 
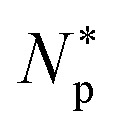
 interchangeably, unless otherwise indicated. Finally, the subscript ‘B’ or ‘H’ will be used to refer to bacteria and host cells, respectively, as in *N*_pB_, *N*_pH_, 
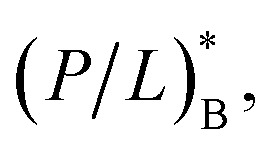
 and 
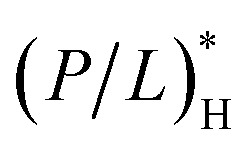
 (see Table 1). Similarly, *C*_B_ is the bacterial cell density and *A*_B_ is the bacterial cell surface area; *a*_B_ and *a*_H_ are the lipid headgroup area of bacterial and host-cell membranes, respectively; the binding energy *w*_B_ and *w*_H_ can be interpreted similarly.

### 2.1 Homogeneous case

**Fig. 2:**
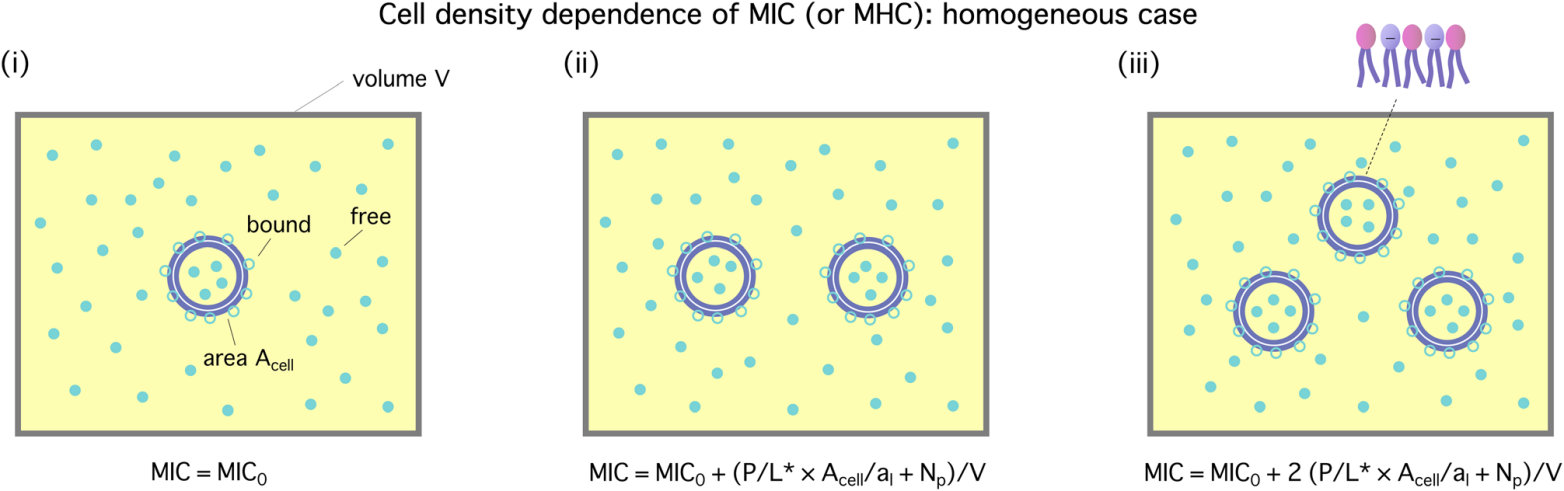
Cell-density dependence of 
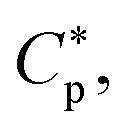
*i.e.*, either MIC or MHC: a homogeneous or noncompetitive case. Cells are represented by two concentric circles and peptides by filled (free or trapped) or unfilled circles (membrane-bound). As the peptide concentration *C*_p_ increases, their surface coverage *P*/*L* (molar ratio of peptides to lipids) also increases and eventually reaches a threshold *P*/L* at 
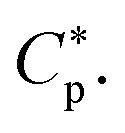
 Even in the single-cell limit shown in (i), 
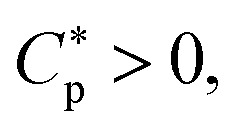
 because of the entropy of peptides, which favors unbinding. Imagine introducing a second cell in (i), converting the system into the one in (ii). The number of peptides the first cell consumed is equal to (*P*/L* × *A*_cell_/*a*_1_ + *N*_p_), where *a*_l_ is the area of each lipid. In order to remain at *P*/L*, the same number of peptides should be supplied. This will raise 
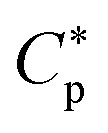
 by *P*/L* × *A*_cell_/*a*_l_ + *N*_p_/*V*, where *V* is the volume of the system: 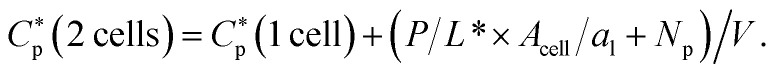 The progression from (i) to (iii) shows that 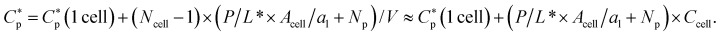 When applied to bacteria, this equation become MIC(*C*_cell_) = MIC_0_ + (*P*/L* × *A*_cell_/*a*_l_ + *N*_p_)*C*_cell_, where MIC_0_ is MIC in the low-cell density limit: *C*_cell_ → 0. Figure adapted with permission from ref. 17. Copyright 2015 American Chemical Society; Reproduced with modifications from ref. 18 with permission from the Royal Society of Chemistry.

Fig. 2 illustrates how 
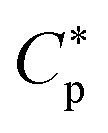
 depends on cell density *C*_cell_ in a homogeneous or noncompetitive case, consisting of either bacteria or host cells. Here, two concentric circles represent cells (membrane bilayers enclosing cells), whereas small circles stand for peptides; if filled ones are free or trapped, unfilled ones are membrane-bound. The fraction of bound peptides is controlled by the balance between entropy and energy^24^ (also see ref. 25). At a low peptide concentration, peptides are mostly free, because of a large entropic penalty for binding even in a single-cell limit (Fig. 2(i)). As the peptide concentration *C*_p_ increases, the balance is swayed toward energy, which favors binding. As a result, the surface coverage of peptides *P*/*L* (molar ratio of bound peptides to lipids) also increases. Eventually, *C*_p_ reaches 
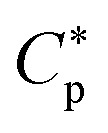
 (either MIC or MHC), at which *P*/*L* = *P*/L*. Even in the single cell limit shown in (i), 
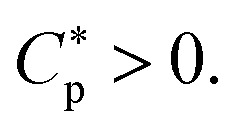


As the cell density increases, different cells compete for peptides. Even though the binding is driven by energy, this competition is entropic in origin and does not involve cell–cell interactions. This is responsible for the cell-density dependence of 
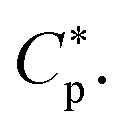
 It can be worked out progressively as shown in Fig. 2. Now imagine introducing a second cell in Fig. 2(i), converting the system into the one in Fig. 2(ii). Because of the presence of the first cell, there will be less peptides for the second one: at 
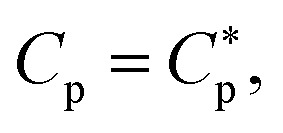
 the number of peptides the first cell consumed is equal to 
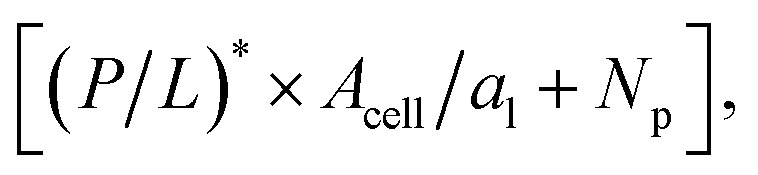
 where *a*_l_ is the area of each lipid; recall *A*_cell_ is the surface area of each cell. The presence of a second cell in (ii) is equivalent to removing [(*P*/*L*)* × *A*_cell_/*a*_l_ + *N*_p_] peptides in (i), which is at 
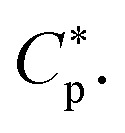
 In order to remain at *P*/L*, an extra number of peptides should be supplied. The required number of peptides is equal to [(*P*/*L*)* × *A*_cell_/*a*_l_ + *N*_p_]. This will raise 
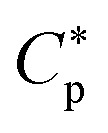
 by [(*P*/*L*)* × *A*_cell_/*a*_l_ + *N*_p_]/*V*, where *V* is the volume of the system:1
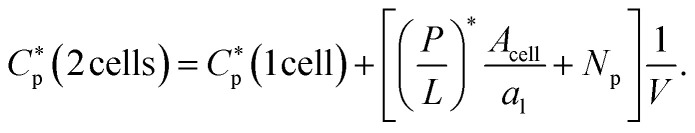


Here, 
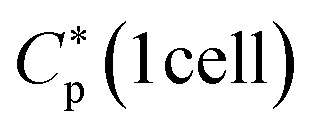
 is either MIC or MHC in the single-cell case.

The progression from (i) to (iii) shows how this analysis can be extended to the *N*_cell_-cell case:2
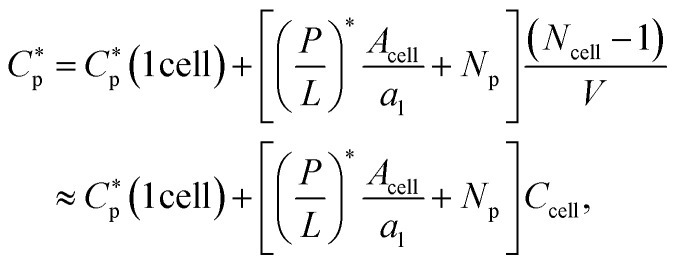
where the second equality holds if *N*_cell_ ≫ 1, as is often the case. This equation can be applied to a homogeneous system of either bacteria or host cells.

Eqn (2) becomes3a
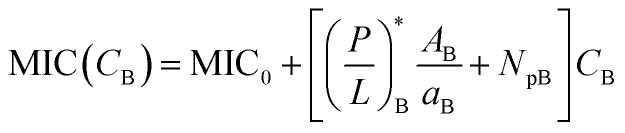
3b
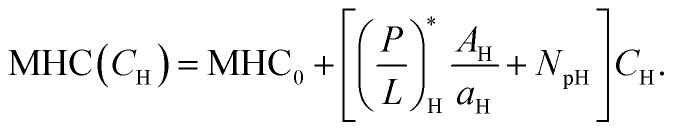


Here MIC_0_ and MHC_0_ are, respectively, the MIC and MHC in the low-cell density limit: 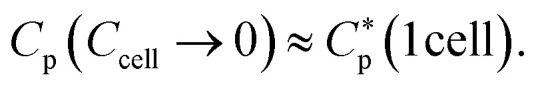


Eqn (3) can be viewed as a function of *C*_cell_: *C*_B_ or *C*_H_. Both the MIC and the MHC increase linearly with the cell density *C*_B_ and *C*_H_, respectively. The slope of the relation in eqn (3), [(*P*/*L*)* *A*_cell_/*a*_l_ + *N*_p_], is the total number of peptides consumed per cell at *P*/*L* = (*P*/*L*)*. This is larger for larger *N*_p_; peptide trapping in cells makes 
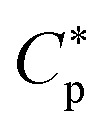
 increase more rapidly with *C*_cell_. The ‘*y*’-axis intercept, either MIC_0_ or MHC_0_, is set by the interaction of peptides with membranes among others (see Section 3). The value of *P*/L* reflects membrane curvature (peptide parameters as well).^19–21^ It is larger for PE (phosphatidylethanolamine)-containing bacterial membranes, which tend to develop a negative curvature. However, this does not change *P*/L* by an order of magnitude. For the peptide melittin, for instance, 
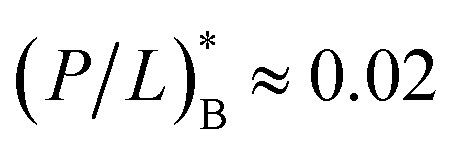
 and 
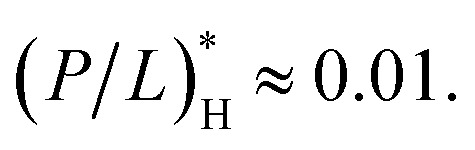
^19–21^

Imagine combining MHC and MIC values obtained separately for homogeneous solutions. The ratio MHC/MIC increases with *C*_H_: the larger *C*_H_ is, the larger the selectivity is. As evidenced below, this does not correctly represent the selectivity in a biological-relevant medium (*e.g.*, a mixture of host cells and bacteria) but tends to overestimate it.

### 2.2 Competitive case

**Fig. 3:**
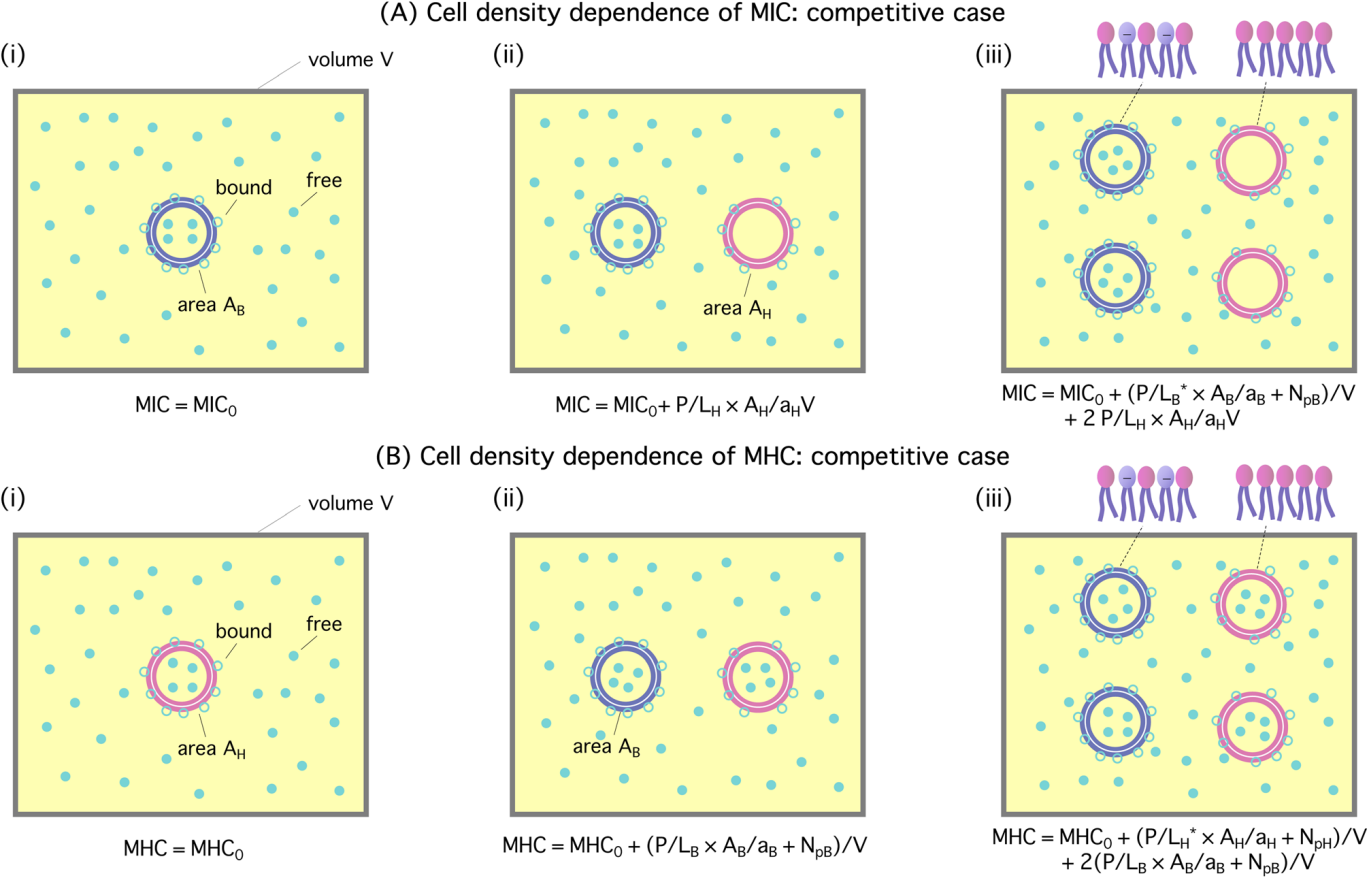
Cell-density dependence of MIC (A) and MHC (B): competitive case. Cells are represented by two concentric circles and peptides by filled (free or trapped) or unfilled circles (membrane-bound); if the blue circles represent bacterial cells, the pink ones stand for host cells. Let *A*_cell_ = *A*_B_ or *A*_H_ be the bacterial or host cell surface area, respectively; *a*_B_ and *a*_H_ the lipid headgroup area of the bacterial or host-cell membranes, respectively; *N*_pB_ and *N*_pH_ the number of trapped peptides in each bacterial and host cell, respectively; (*P*/*L*)_B_ and (*P*/*L*)_H_ are the molar ratio of bound peptides to lipids on the bacterial and host-cell membranes, respectively. (A) The progression from (i) to (iii) suggests that 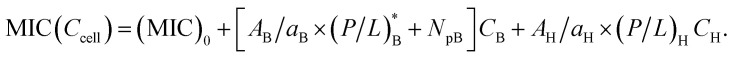 (B) Using a similar line of reasoning, we arrive at 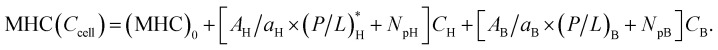 Figure adapted with permission from ref. 17. Copyright 2015 American Chemical Society; Reproduced with modifications from ref. 18 with permission from the Royal Society of Chemistry.

The homogeneous-case analysis in Fig. 2 can be extended to a mixture of bacterial and host cells, referred to as a competitive case, as shown in Fig. 3. If the concentric circles in blue represent bacterial cells, the pink ones stand for the host cells. Fig. 3(i) shows a single bacterial cell at the MIC. The introduction of a host cell in Fig. 3(ii) will reduce the amount of peptides for the bacterial cell. The extra number of peptides to maintain *C*_p_ at the MIC is equal to [(*P*/*L*)_H_ × *A*_H_/*a*_H_ + *N*_pH_]; similarly, in Fig. 3(iii), the number of peptides that should be added is 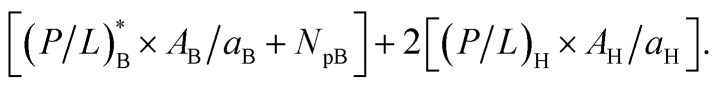


The progression from (i) to (iii) suggests that4a
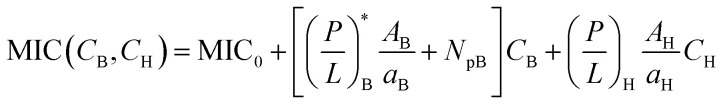
4b
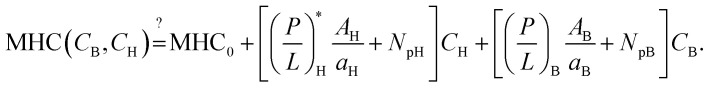


If *N*_p_ is set to zero as for model membranes, the second equation in eqn (4) can be obtained from the first one by swapping the role of bacteria with that of host cells. Here (*P*/*L*)_H_ in eqn (4a) is the surface coverage of peptides on the host cells evaluated at *C*_p_ = MIC, whereas (*P*/*L*)_B_ in eqn (4b) is the surface coverage of peptides on bacteria evaluated at *C*_p_ = MHC.

Note that these two lines of equations in eqn (4) are not fully symmetric with respect to the exchange in role between host cells and bacteria for the obvious reason: as *C*_p_ increases, the MIC will be reached first. This explains why the last term in eqn (4a) does not contain *N*_pH_. In other words, 
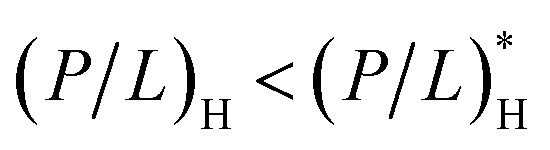
 in eqn (4a). In contrast, 
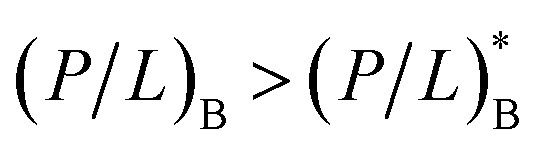
 in eqn (4b). As a result, over a sizeable *C*_p_ range, the peptide under consideration is active against bacteria only and is thus selective.

Also, eqn (4b) needs to be understood with caution. Beyond 
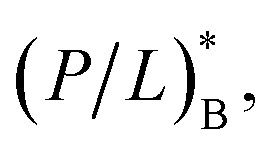
 some of bound peptides start to rupture the membranes by forming pores, for instance. The last term in these equations may be interpreted as the total amount of bound peptides whether on the membrane surface or in pores. As a result, the binding energy *w*_B_ needs to be interpreted accordingly. As it turns out, the term inside […] in eqn (4) is dominated by *N*_p_ (see below). Furthermore, *w*_H_, which governs peptide binding and influences (*P*/*L*)_H_, is not constant but can vary with (*P*/*L*)_H_. The main source of this dependence is the electrostatic interaction between bound peptides. But this dependence is generally weak, since the distance between bound peptides for (*P*/*L*) ≤ (*P*/*L*)* ≈ 0.01 is typically larger than the Debye screening length, *r*_D_, beyond which the electrostatic interaction is exponentially screened.^25^ At (*P*/*L*)* = 0.01, the typical distance between the adjacent peptides is 
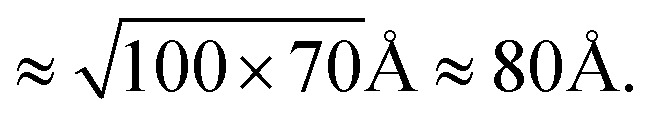
 This is appreciably larger than the screening length under physiological conditions (*e.g.*, in the presence of 100 mM of monovalent salts): *r*_D_ ≈ 10 Å. Finally, in eqn (4b), *N*_pB_ is the number of trapped peptides in each cell above the MIC. The value of this parameter will eventually be determined by chemical equilibrium between trapped peptides and those on the membrane or in the bulk. The energetics of this is unknown and can be influenced by a number of factors such as peptide's interaction with cellular components and crowding in the cell. As mentioned in Section 2, in our consideration, we ignore this complexity and approximate *N*_pB_ in eqn (4b) by 
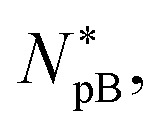
*i.e.*, *N*_pB_ at MIC.

It is worth noting that eqn (3) and (4) are a special case of the following relations:5a
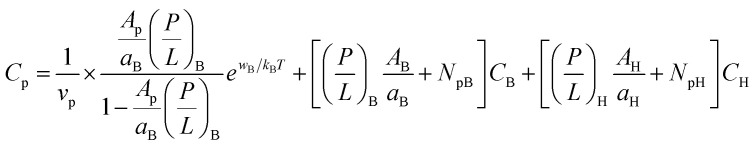
5b
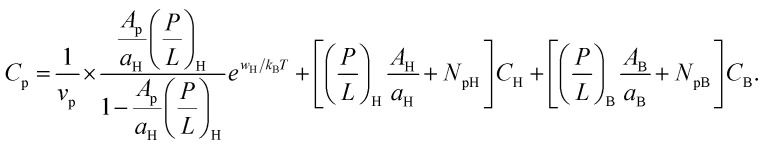
Here, *v*_p_ is the volume occupied by each peptide in the bulk and *A*_p_ is the peptide area on the membrane surface. The first term in each line is inspired by eqn (11); recall *w*_B_ and *w*_H_ are, respectively, the binding energy of a given peptide on bacterial and host-cell membranes (for details, see the ESIof ref. 17 or Section 3). If evaluated at 
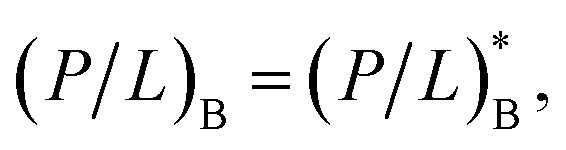
 the first term on the right hand side of eqn (5a) is MIC_0_; the first term in eqn (5b) can be interpreted similarly. Strictly speaking, both *w*_B_ and *w*_H_ have a weak dependence on *P*/*L*. At the relevant range of *P*/*L* around *P*/L*, however, this dependence can be neglected as discussed above.

The meaning of *N*_p_ in eqn (5) is somewhat different from that in eqn (4). As noted above, eqn (5) is more general in the sense that *C*_p_ on the left hand side does not have to be equal to 
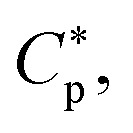
 which is either MIC or MHC. As a result, *N*_p_ in eqn (5) varies with *P*/*L* and is generally different from 
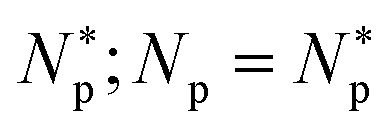
 for (*P*/*L*) = (*P*/*L*)* and *N*_p_ = 0 for (*P*/*L*) < (*P*/*L*)* Accordingly, *N*_pH_ = 0 in eqn (5a), when *C*_p_ = MIC (<MHC). Eqn (5a) then reduces to the MIC expression in eqn (4a). Similarly, eqn (5b) becomes the MHC expression in eqn (4b) in an appropriate limit.

For given values of *C*_p_ and cell density (*C*_B_ and *C*_H_), the two equations in eqn (5) can be solved simultaneously for *P*/*L*: (*P*/*L*)_B_ and (*P*/*L*)_H_. Initially, we set *N*_p_ = 0 and increase *C*_p_ gradually from zero. At some value of *C*_p_, (*P*/*L*)_B_ reaches 
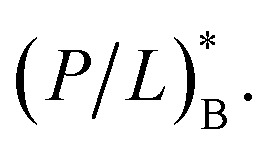
 The resulting value of *C*_p_ with *N*_pB_ set to 
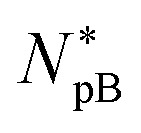
 is the MIC. We then increase *C*_p_ further until 
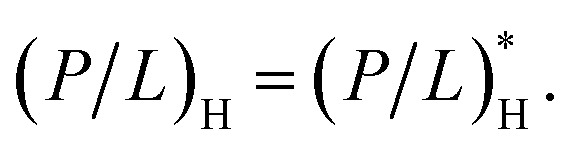
 The resulting *C*_p_ with 
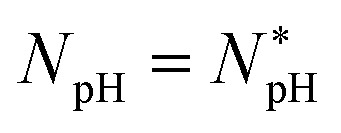
 is the MHC. In this step, (*P*/*L*)_B_ in eqn (5) is larger than 
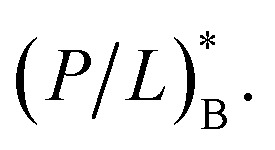
 In reality, pore formation in bacterial membranes can complicate the energetics of peptide binding to the membrane. But this complication will not change the MHC in any significant way, since (*P*/*L*)_B_ (*A*_B_/*a*_B_) ≪ *N*_pB_ at or above the MHC, as discussed in ref. 16 (also see below); the main source of inoculum effects is the trapping of peptides in cells rather than peptide adsorption to membranes. For model membranes, however, this reasoning is not applicable. In our coarse-grained model, all the details governing peptide binding are subsumed into the parameter *w* (*w*_B_ and *w*_H_). As noted above, *w* has a weak dependence on *P*/*L* and can also be influenced by pore formation. In a Langmuir-type model such as the one employed here, *w* is often approximated by its representative value. With a similar spirit, we will use a standard value of *N*_p_, as discussed in Section 3.

Let's analyze the relative significance of peptide trapping in determining the cell-density dependence of MIC or MHC. For this, we essentially repeat the earlier analysis in ref. 16. Compare the two terms with each other inside […] in eqn (4): the number of membrane-bound peptides and the number of absorbed peptides per cell. For the representative bacterium *E. coli*, *A*_B_ ≈ 12 μm^2^, which is twice the area of the inner or outer layer of the cytoplasmic membrane.^16,17^ Since *a*_B_ ≈ *a*_H_ ≈ 70 Å^2^, *A*_B_/*a*_B_ ≈ 1.7 × 10^7^. For the peptide melittin, 
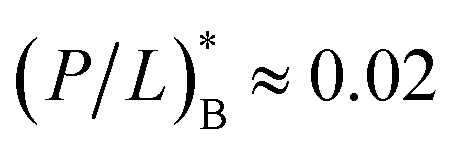
 and 
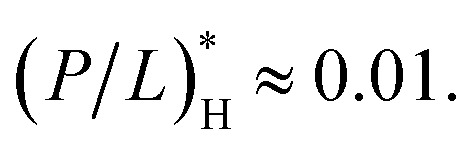
^19–21^ We thus find 
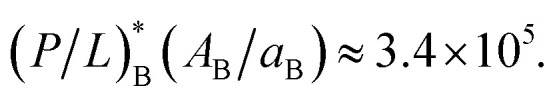
 This number is much smaller than *N*_pB_ ≈ 10^7^ to 10^8^.^14^ For the outer *E. coli* membrane, 
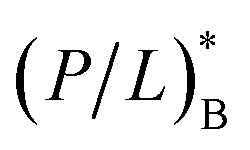
 is several fold larger,^10,26^ but this does not change the picture. For human red blood cells as representative host cells, *A*_H_ ≈ 17*A*_B_ and *A*_H_/*a*_H_ ≈ 2.9 × 10^8^. As a result, we obtain 
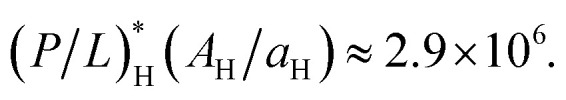
 This is smaller than *N*_pH_ ≈ 10^7^.^12,13^ The main source of inoculum effects is the trapping of peptides inside dead cells at or above (*P*/*L*)*.

The analysis above implies that only the last term in eqn (4a) has a noticeable, explicit dependence on the binding energy *w*_H_ for given MHC_0_. As a result, the MIC in eqn (4a) can be sensitive to *w*_H_, whereas the MHC in eqn (4b) is not. For similar reasons, both the MIC and the MHC in eqn (4) and (3) are not sensitive to *w*_B_ for a fixed value of MIC_0_. For the homogeneous case in eqn (3), none of the MIC and the MHC is “explicitly” sensitive to *w*_B_ or *w*_H_.

Similarly to what was observed in the homogenous case in Section 2.1, peptide trapping in cells (the main inoculum effect) makes 
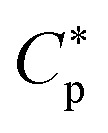
 increase more rapidly with *C*_cell_. It makes steeper the slope of a 
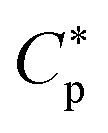
 curve *versus C*_cell_.

### 2.3 Limiting cases

It proves instructive to take some mathematical limits and simplify eqn (4). First, consider the case *C*_B_ = *C*_H_. In the low cell-density limit, *i.e.*, *C*_B_ = *C*_H_ → 0, the MIC and MHC in eqn (4) reduce to MIC_0_ and MHC_0_, respectively, as there is no competition between different cells (or membranes) to bind peptides. As a result, the distinction between the competitive and noncompetitive cases disappears in this limit.

In the high-cell-density case, for simplicity, let's assume that *A*_B_ = *A*_H_ and *N*_p_ = 0, as is often the case for lipid bilayers, and *a*_B_ = *a*_H_ ≈ 70 Å^2^, which is a good approximation (if *A*_H_ ≠ *A*_B_, this analysis is applicable to the case: *A*_B_*C*_B_ = *A*_H_*C*_H_). The competitive selectivity, MHC/MIC, becomes cell-density independent: 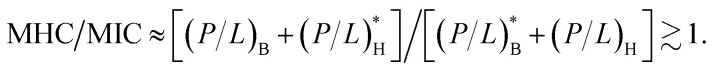 To understand the origin of the inequality, note that (*P*/*L*)_B_ in the numerator is larger than 
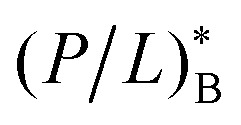
 in the denominator, whereas (*P*/*L*)_H_ in the denominator is smaller than 
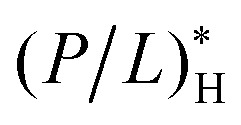
 in the numerator. Thus MHC/MIC in this limit will get saturated at some constant larger than 1.

In the noncompetitive case with *C*_B_ = *C*_H_, however, the ratio MHC/MIC approaches the following constant: 
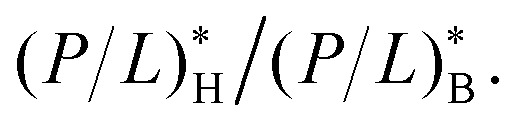
 The threshold *P*/*L* is better known for lipid bilayers mimicking cell membranes than for cell membranes. As noted in Section 2.1, because of the presence of PE (phosphatidylethanolamine) in bacterial cell-membrane mimics, 
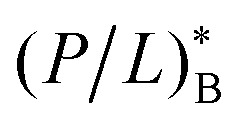
 is somewhat larger than 
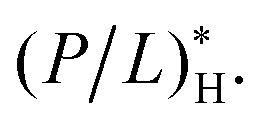
 In the large cell-density limit in the noncompetitive case, we thus have MHC/MIC ≲ 1. There is a noticeable difference between the competitive and noncompetitive cases in the large cell-density limit; the selectivity is higher in the former case.

**Fig. 4:**
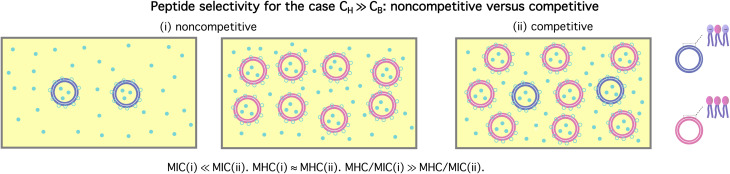
Peptide selectivity for a noncompetitive (homogeneous) (i) *versus* competitive (heterogeneous) case (ii). It is assumed that *C*_H_ ≫ *C*_B_. In this case, whether the selectivity is measured noncompetitively (i) or competitively (ii) has a profound impact on the selectivity. It can be excessively overestimated in the noncompetitive case (i) with reference to the corresponding competitive case (ii), since the MIC is much larger for the latter case. The opposite is true if *C*_H_ ≪ *C*_B_. Figure adapted with permission from ref. 17. Copyright 2015 American Chemical Society; Reproduced with modifications from ref. 18 with permission from the Royal Society of Chemistry.

If *C*_H_ ≫ *C*_B_, eqn (4) can be simplified as MIC ≈ (MIC)_0_ + *A*_H_/*a*_H_ × (*P*/*L*)_H_*C*_H_ and 
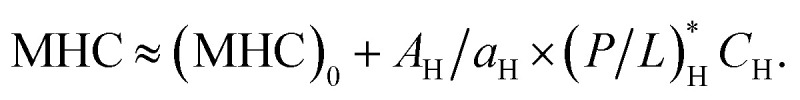
 Note that the MIC in this case is much larger than the MIC for the corresponding bacteria-only case and the MHC here is approximately equal to the MHC for the corresponding host-cell-only case, as illustrated in Fig. 4. Accordingly, the ratio MHC/MIC is roughly independent of *C*_B_ and approaches a constant of order 1, as *C*_H_ → ∞ (while *C*_B_ is held fixed).

Imagine combining two sets of data: one set for bacteria only and one set for host cells only, *i.e.*, two homogeneous cases in eqn (3). If *C*_H_ ≫ *C*_B_, MHC/MIC → ∞ as *C*_H_ → ∞. This limiting behavior in the homogeneous case is opposite to the one obtained for the corresponding competitive case (see Fig. 4). It explains how the selectivity can be excessively overestimated.

When *N*_p_ ≠ 0 and *A*_B_ ≠ *A*_H_, our analysis should reflect these inequalities. But the difference caused by them is often quantitative rather than qualitative, as evidenced in Section 4.

A full analysis of eqn (4) is involved. As discussed earlier,^16^ in some relevant limits, we can simplify eqn (4) (see ref. 16). This is particular the case for *C*_H_ ≫ *C*_B_ as in whole blood. In this case, eqn (4) can be approximated as6a
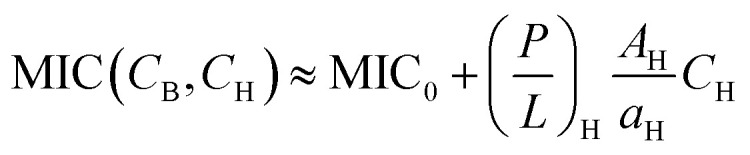
6b
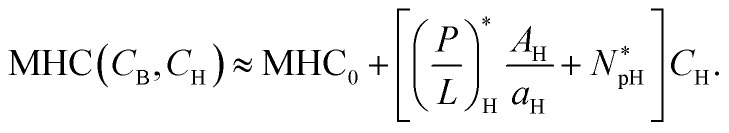
Here, (*P*/*L*)_H_ is to be evaluated at *C*_p_ = MIC.

Notice the obvious difference the competitive MIC in eqn (6a) and the noncompetitive one in eqn (3a). As discussed earlier in Section 2.3 and in Fig. 4, for *C*_H_ ≫ *C*_B_, the competitive MIC is much larger than the noncompetitive one. In contrast, the MHC is approximately the same for both cases. This results in much larger selectivity in the noncompetitive case compared to the corresponding competitive case. This finding is consistent with the analysis above with *N*_p_ set to zero.

For the case *C*_H_ ≫ *C*_B_, the ratio MHC/MIC becomes7
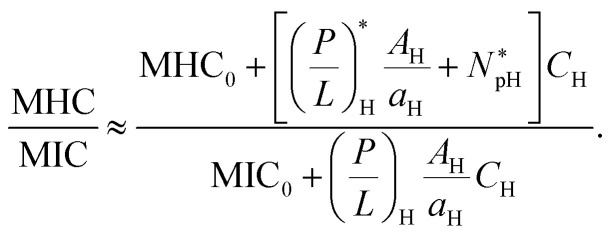
Eqn (7) suggests that peptide trapping in the host cells enhances peptide selectivity; it works in favor of the host cells. This is a natural consequence of the MHC that increases with 
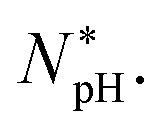
 Since the second term inside […] in the numerator of eqn (7) is larger than the first term roughly by an order of magnitude (see Section 2.2), the effect of peptide trapping on the selectivity is up to about 10-fold.

So far, we have used a simple biophysical picture, based on Fig. 2–4 to explore how peptide selectivity depends on cell density (*C*_B_ or *C*_H_) and on the way it is measured (*i.e.*, competitive *versus* noncompetitive). The *y*-intercepts, MIC_0_ and MHC_0_, may be considered as fitting parameters. They can also be related to more microscopic parameters. In the next section, we recapture the main results in this section; we then relate MIC_0_ and MHC_0_ to the biophysical parameters of peptides and membranes.

## 3. Langmuir binding model

In this section, using a Langmuir-type model for molecular binding,^25^ we derive the main results presented in Section 2 and relate MIC_0_ and MHC_0_ to the biophysical parameters of peptides and membranes. Note that such a model was already considered recently.^17,18^ Here, we recapture the essence of this consideration and generalize it to include peptide trapping in a cell. It suffices to focus on the homogeneous case, since the dependence of peptide activity on cell density in the competitive case is already obvious from eqn (4).

In this model, peptides are either “free” (in the bulk) or “bound”; bound peptides are further classified as adsorbed to the cell surface or trapped inside a cell (see Fig. 2); trapped ones can bind to intracellular components. Initially, peptide binding occurs on the outer membrane layer or the outmost one in the case of Gram-negative bacteria. Adsorbed peptides will be eventually symmetrically distributed between the two layers after or even prior to membrane rupture^27,28^ (also see ref. 17). For simplicity, we ignore peptide trapping below 
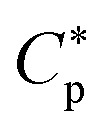
 within typical experimental time scales. Indeed, it was shown that a large amount of trapped peptides were observed in dead bacterial cells, but not in dividing cells.^14^ At and beyond 
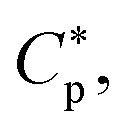
 the amount of bound peptides is determined by chemical equilibrium between free and bound states.

Let *w* and *u* be the adsorption and trapping energy, respectively. The value of *w* is typically more negative for bacterial membranes containing a large fraction of anionic lipids. It is worth noting that *w* is an effective parameter in which the effect of lipid demixing and peptide–peptide interactions on the membrane surface are subsumed (see ref. 18 for details). Similarly, *u* takes into account the interactions of trapped peptides with intracellular components as well as their mutual interactions inside the cell; it is also influenced by molecular crowding in the cell.^29,30^

Let *C*_p_ be the total concentration of peptides whether free or bound, *σ*_p_ [=(*P*/*L*)/*a*_l_] the planar density of adsorbed peptides and *A*_p_ the area occupied by a bound peptide; *n*_p_ the number density of trapped peptides, and *v*_p_ the volume of each peptide; *n*_p_ = 0 when *P*/*L* < *P*/L* and *n*_p_ = *N*_p_/*V*_cell_ when *P*/*L* = *P*/L*, where *V*_cell_ is the volume of each cell. In our Langmuir model, the chemical potential of bound peptides *μ*_bound_ at and above *P*/L* can readily be obtained as8
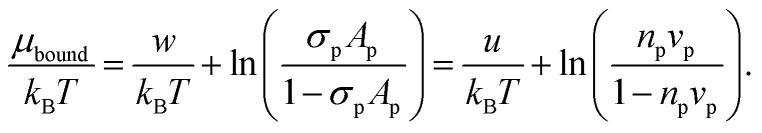
Here and below, *k*_B_ is the Boltzmann constant and *T* the temperature. The logarithmic term is related to the number of ways in which bound peptides are distributed on the membrane surface or inside the cell. The second equality holds in chemical equilibrium between adsorbed and trapped peptides.

The chemical potential of free peptides is9
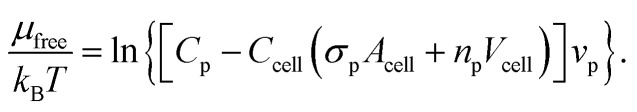
Note that the expression inside […] is the concentration of free peptides and the term inside (…) is the inoculum size.

By equating the two chemical potentials in eqn (8) and (9), we obtain10a
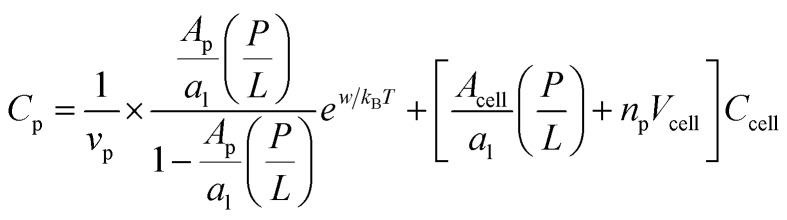
10b
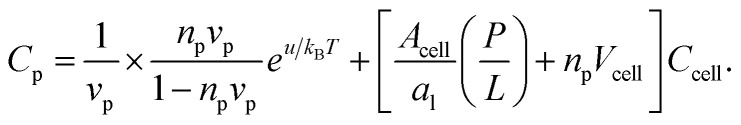
In this final expression, we eliminated *σ*_p_ in favor of *P*/*L via* the relation *σ*_p_*a*_l_ = *P*/*L* (with *a*_l_ as the lipid head-group area). In the absence of peptide trapping in cells (or below 
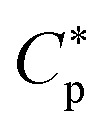
), eqn (10a) with *n*_p_ = 0 describes chemical equilibrium between free and adsorbed peptides; eqn (10b) becomes irrelevant.

At 
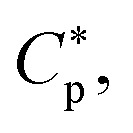
*i.e.*, either MIC or MHC,11a
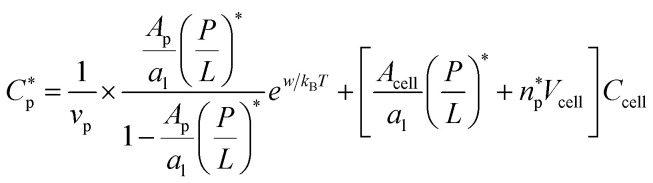
11b
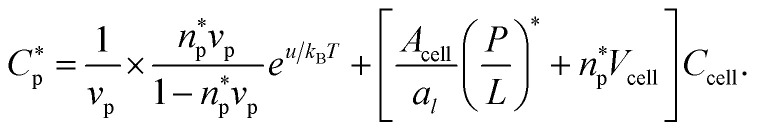
Here 
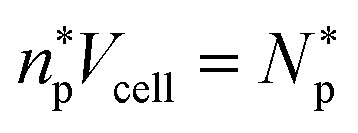
 is the (average) number of peptides trapped in each cell at 
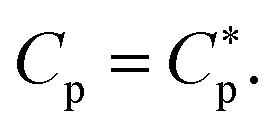


Comparison between eqn (11) and (3) leads to the following relation12a
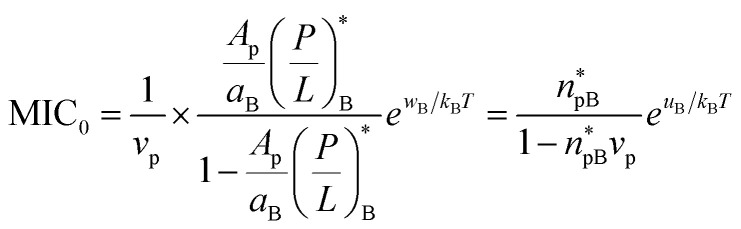
12b
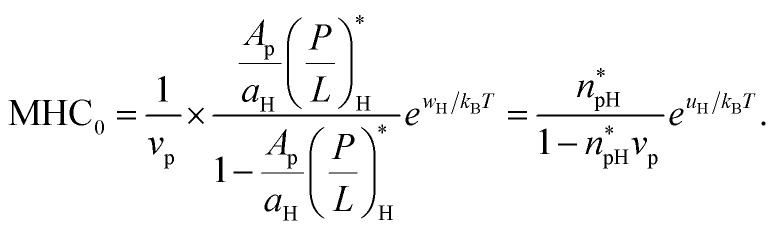
Here the subscript ‘B’ and ‘H’ refer to bacteria and host cells, respectively. Both MIC_0_ and MHC_0_ are exponentially sensitive to *w* or *u* but they are not as sensitive to other quantities. This energy scale is the main origin of peptide selectivity. The results in eqn (12) can be used in eqn (4) (competitive) or in eqn (3) (noncompetitive).

It is worth mentioning that we will not attempt to solve eqn (10) for *n*_p_, partly because the energetics involved in peptide trapping (*i.e.*, *u*) is not well known. Instead, we will use suitable values of 
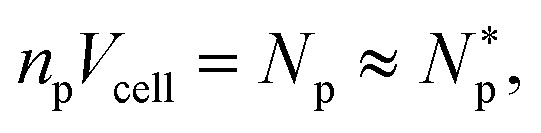
 the number of peptides trapped in each cell, inspired by recent experiments.^12–14^ With this simplification, eqn (10) can readily be extended to the competitive case shown in Fig. 3. The cell-density dependence of 
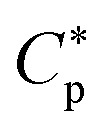
 is already obvious in light of the discussion in Section 2.2; one can readily write down eqn (5).

## 4. Results

In this section, we present the results for peptide activity and selectivity obtained for model membranes (Section 4.1) and cells (Section 4.2). Recall that one of the main differences between the two comes from peptide trapping in the latter case. As detailed below, MIC_0_ and MHC_0_ are chosen differently for the two cases. If calculated values of these quantities are used for model membranes, they are chosen appropriately for cells.

### 4.1 Membrane selectivity

Following Section 2, we first present our results for peptide activity and selectivity without taking into account peptide trapping using peptide parameters relevant for a melittin-like peptide:^17,18^
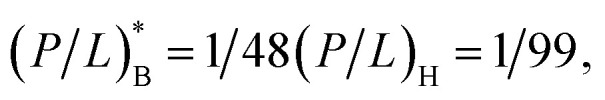
^19–21^*v*_p_ = 33^3^ Å^3^, and *A*_p_ = 400 Å^2^.^17,18^ For this peptide, *w* was mapped out for model membranes, mimicking bacterial and host-cell membranes: *w*_B_ = −16.6*k*_B_*T* and *w*_H_ = −6.72*k*_B_*T*.^18^ Also, *a*_B_ = 71 Å^2^ (*a*_l_ for bacterial membranes), *a*_H_ = 74 Å^2^ (*a*_l_ for host-cell membranes),^19–21^*A*_B_ = 1.2 ×10^9^ Å^2^ = 12 μm^2^ (suitable for *E. coli*), and *A*_H_ = *A*_B_ or *A*_H_ = 17*A*_B_ (as for human red blood cells).^17^ Note here that this value of *A*_B_ is two times the surface area of *E. coli* (≈6 μm^2^).^31^ This is to reflect the symmetrical binding of peptides on the inner and outer layers of the cytoplasmic membrane, as discussed in Section 3. Finally, we set *N*_p_ = 0 as expected for model membranes. In reality, *N*_p_ ≠ 0 at or beyond (*P*/*L*)*. But practically, it can be set to zero, since the majority of peptides would remain ‘free’; trapped peptides in model membranes are outnumbered by those in the bulk.

**Fig. 5:**
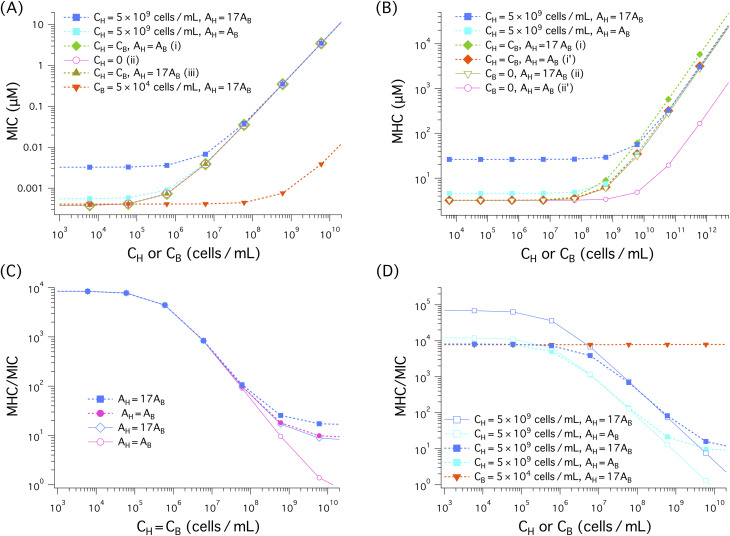
Cell (membrane) density dependence of MIC, MHC, and MHC/MIC for the noncompetitive and competitive cases, represented by solid lines with unfilled symbols and dashed lines with filled symbols, respectively. When *C*_H_ (*C*_B_) is held fixed, the ‘*x*’ axis represents *C*_B_ (*C*_H_); for the case *C*_H_ = *C*_B_, it stands for both *C*_H_ and *C*_B_. We have chosen the parameter as follows: the bacterial cell surface area *A*_B_ = 12 μm^2^ (suitable for *E. coli*); the host cell surface area *A*_H_ = *A*_B_ and *A*_H_ = 200 μm^2^ ≈ 17 × *A*_B_ (as for human red blood cells); *a*_B_ = 71 Å^2^ and *a*_H_ = 74 Å^2^; 
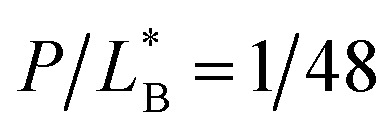
 and 
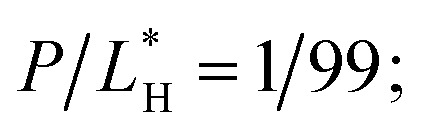
^19–21^*v*_p_ = 33^3^ Å^3^ and *A*_p_ = 400 Å^2^;^17,18^*w*_B_ = −16.6 *k*_B_*T* and *w*_H_ = −6.72 *k*_B_*T*^18^ as for the peptide melittin. (A)–(B) In all cases, both MICs and MHCs increase with increasing *C*_H_ or *C*_B_, as expected from eqn (4a). Also, the presence of a large amount of hot-cell membranes (*C*_H_ = 5 × 10^9^ cells per mL) raises both the MIC and the MIC, almost by an order of magnitude for the case *A*_H_ = 17*A*_B_ as long as *C*_H_ ≫ *C*_B_. There is no essential difference between the three cases labelled as (i), (ii), and (iii) in the legend in (A): the presence of an equal amount of host-cell membranes (*C*_H_ = *C*_B_) or the value of *A*_H_ does not influence the MIC in any noticeable way. As shown in (B), the MHC is larger for larger *A*_H_ (*i.e.*, *A*_H_ = 17*A*_B_). For this, compare a curve obtained with *A*_H_ = *A*_B_ with the corresponding one obtained with *A*_H_ = 17*A*_B_. Also, the MHC curve labelled as (i) lies somewhat above the one labelled as (ii), both obtained with *A*_H_ = 17*A*_B_. In this case, the presence of an equal amount of bacterial membranes (*C*_H_ = *C*_B_) increases slightly the MHC. When *A*_H_ = *A*_B_ represented as (i’) and (ii’), however, it has a more appreciable impact on the MHC. The competitive MHC in the presence of 5 × 10^4^ cells per mL of bacterial membranes with *A*_H_ = 17*A*_B_ is almost identical to the corresponding noncompetitive one (*i.e.*, *C*_B_ = 0) (data not shown for simplicity). The selectivity in (C), as measured by MHC/MIC, decreases as the membrane density increases; in both competitive and noncompetitive cases, we chose *C*_H_ = *C*_B_. The difference between the competitive and noncompetitive cases becomes obvious when the cell density is ≳ 10^8^ cells per mL, in which the selectivity is higher for the former case. Also the selectivity is higher for the larger *A*_H_ case as long as *C*_B_ = *C*_H_ ≳ 10^8^ cells per mL. In (D), except for the red dashed curve with inverted filled triangles, *C*_H_ = 5 × 10^9^ cells per mL but *C*_B_ varies. Similarly to what the graph in (C) suggests, the selectivity in (D) decreases as *C*_B_ decreases. Compared to the competitive case represented by the blue dashed curve with filled squares, the corresponding noncompetitive case overestimates the selectivity by about one order of magnitude at a low *C*_B_ range of *C*_B_ ≲ 10^5^ cells per mL. The red dashed line with inverted triangles obtained with *C*_B_ = 5 × 10^4^ cells per mL is nearly flat in the *C*_H_ range shown.

We have solved eqn (3) for the noncompetitive case and eqn (4) for the competitive case (both together with eqn (12)). This is equivalent to solving eqn (5) for *P*/*L* at and found *C*_p_ at which *P*/*L* is equal to *P*/L*, as discussed below eqn (5). In Fig. 5, the resulting 
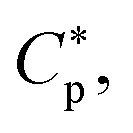
 either MIC or MHC, as well as the ratio MHC/MIC are shown as a function of cell density: *C*_B_ or *C*_H_. When *C*_H_ (*C*_B_) is held fixed, the *x* axis represents *C*_B_ (*C*_H_); for the case *C*_H_ = *C*_B_, it indicates both *C*_H_ and *C*_B_. If the competitive cases are represented by dashed lines with filled symbols, the noncompetitive ones are described by solid lines with open symbols.

The results in Fig. 5(A) and (B) suggest that both MICs and MHCs increase with increasing cell density (*C*_H_ or *C*_B_), as expected from eqn (3) and (4). For *A*_H_ = 17*A*_B_, the presence of a large amount of host-cell membranes (*C*_H_ = 5 × 10^9^ cells per mL) raises the MIC by an order of magnitude as long as *C*_B_ ≲ 5 × 10^5^ cells per mL, compared to the case *C*_H_ = 0; for *A*_H_ = *A*_B_, however, its impact on the MIC appears to be minor. There is no essential difference between the three cases in (A): (i) *C*_H_ = *C*_B_, *A*_H_ = *A*_B_, (ii) *C*_H_ = 0, *A*_H_ = *A*_B_, and (iii) *C*_H_ = *C*_B_, *A*_H_ = 17*A*_B_ (labelled as (i), (ii), (iii), respectively, in the legend); in these cases, the MIC is insensitive to the presence of an equal amount of host-cell membranes or the value of *A*_H_. As *C*_B_ increases, the MIC curves eventually collapse onto each other. In this case, it is dominated by the *C*_B_-dependent term in eqn (4a). This applies to all the curves shown except the one in tangerine for which *C*_B_ is held fixed.

As shown Fig. 5(B), in the presence of a large amount of host-cell membranes (*C*_H_ = 5 × 10^9^ cells per mL), the MHC obtained with *A*_H_ = 17*A*_B_ is about ten times larger than MHC_0_ ≈ 3 μM (*i.e.*, the *y*-intercept of the curve labelled as (ii) or (ii’)), as long as *C*_B_ ≲ 10^8^ cells per mL. Similarly, in the other cases shown, the MHC is larger for larger *A*_H_ = 17*A*_B_ than for *A*_H_ = *A*_B_, as long as *C*_H_ ≳ 10^8^cells per mL. For this, compare a curve obtained with *A*_H_ = *A*_B_ with the corresponding one obtained with *A*_H_ = 17*A*_B_ (*e.g.*, the curves labelled as (i) and (i′) or those labelled as (ii) and (ii′)). The difference between (i) and (i′) seems somewhat minor, but the difference between (ii) and (ii′) (in the absence of bacterial membranes) is pronounced. Also, the MHC curve labelled as (i) lies somewhat above the one labelled as (ii), both obtained with *A*_H_ = 17*A*_B_. In this case, the presence of an equal amount of bacterial membranes (*C*_H_ = *C*_B_) increases slightly the MHC. When *A*_H_ = *A*_B_ (see the curves labelled as (i′) and (ii′)), however, the presence of an equal amount of bacterial membranes has a more appreciable impact on the MHC. In other words, the presence of an equal amount of bacterial cells increases the MHC more effectively when *A*_H_ = *A*_B_. This is consistent with eqn (3) or 4, which suggests that the MHC is more sensitive to *C*_B_ if *A*_H_ is smaller. The presence of 5 × 10^4^ cells per mL of bacterial membranes (*A*_H_ = 17*A*_B_) does not have any noticeable impact on the MHC (the data not shown for simplicity).

The MIC and MHC results in Fig. 5(A) and (B) suggest that the presence of an equal amount of bacterial membranes influences MHCs more effectively than the presence of an equal amount of host cell membranes influences MICs. For this, compare the two curves labeled as (i) *C*_H_ = *C*_B_, *A*_H_ = *A*_B_ and (ii) *C*_H_ = 0, *A*_H_ = *A*_B_ in (A) as well as those labelled as (i’) *C*_H_ = *C*_B_, *A*_H_ = *A*_B_ and (ii′) *C*_B_ = 0, *A*_H_ = *A*_B_ in (B). The two in (A) tend to collapse onto each other, whereas in (B) the curve obtained with *C*_B_ = 0, *A*_H_ = *A*_B_ falls well below the other one. This difference can be attributed to the stronger binding of peptides to bacterial membranes.

In the competitive case with an excessive amount of host cells (*C*_H_ = 5 × 10^9^ cells per mL), however, the MIC in Fig. 5(A) and the MHC in Fig. 5(B) are much larger than in the other cases as long as *C*_B_ ≪ *C*_H_. This is consistent with what eqn (4) suggests: the presence of a large amount of host cells increases both the MIC and the MHC (see the relevant discussion in Section 2.3). These equation also suggest that the MIC or the MHC is generally larger for *A*_H_ = 17*A*_B_ than for *A*_H_ = *A*_B_, unless the *A*_H_-independent terms dominate. For this, compare the two curves in blue and cyan in (A) or (B), for instance. As discussed in Section 2, increasing *A*_H_ is equivalent to increasing *C*_H_. This explains the observation of larger MIC and MHC values for larger *A*_H_.^16,17^

The ratio MHC/MIC measures peptide selectivity. Our results for this ratio are shown in Fig. 5(C) and (D). In (C), *C*_B_ = *C*_H_; in (D), except for the red dashed line, *C*_H_ = 5 × 10^9^ cells per mL but *C*_B_ is allowed to vary. In all cases, the selectivity decreases (or remains flat), as the cell density increases as discussed in Section 2. In (C), the difference between the competitive and noncompetitive cases for *A*_H_ = *A*_B_ becomes obvious when the cell density is ≳ 10^8^ cells per mL, in which the selectivity is higher for the former case. This is correlated with the observation that the MHC is higher for the competitive case in this range of cell density as shown in Fig. 5(B). Also, the selectivity is higher for the larger *A*_H_ case.

The competitive cases in (D), except for the red dashed line, contain a large amount of host cells (*C*_H_ = 5 × 10^9^ cells per mL) in addition to bacterial cells with variable *C*_B_. In the noncompetitive measurement, MHCs obtained with the choice *C*_H_ = 5 × 10^9^ cells per mL were combined with MICs. Similarly to what the graphs in (C) suggests, the selectivity in (D) decreases as *C*_B_ increases. However, the selectivity in the noncompetitive case is overestimated compared to the corresponding competitive case, as long as *C*_B_ ≲ 10^7^ to 10^8^ cells per mL ≪ *C*_H_. For the large *A*_H_ case, it is overestimated by up to an order of magnitude. When *C*_B_ is held fixed at *C*_B_ = 5 × 10^4^ cells per mL, the (competitive) selectivity remains roughly flat in the *C*_H_ range shown.

This finding is well aligned with the view that the selectivity measured in a noncompetitive manner (with *C*_H_ ≫ *C*_B_) can be an experimental illusion.^11^ This is not the case for the competitive selectivity. Even in the presence of a large amount of host cells, the selectivity measured in a competitive environment is not an experimental artifact. It just reflects the cell-density dependence of the selectivity, presented in Section 2.

### 4.2 Cell selectivity: inoculum effects

We have solved eqn (4) with realistic choices of *N*_p_ and mapped out various scenarios for peptide activity and selectivity. One of the challenges in this effort is that the parameters in these equations are not well known for real cells. In particular, *w*_B_ for Gram-negative bacteria is also influenced by the peptide interaction with their outer membrane (OM); recall that this is an effective parameter, in which microscopic details (*e.g.*, peptide charge, peptide interaction with the OM) are subsumed (see Section 3). This quantity has only recently been mapped out theoretically for the interaction of melittin-like peptides with model membranes.^18^ For the reasons explained in Section 2, however, the dependence of peptide activity on *w*_B_ is reflected mainly through MIC_0_. Furthermore, the MIC and the MHC in the homogeneous case in eqn (3) do not depend sensitively on *w*_B_ or *w*_H_ for given MIC_0_ and MHC_0_.

Here we do not attempt to calculate the effective binding energy *w* (either *w*_B_ or *w*_H_) for real cells and use it in the computation of MIC_0_ and MHC_0_. Instead, we start with conveniently-chosen but biophysically-relevant values of MIC_0_ and MHC_0_: MIC_0_ = 1 μm and MHC_0_ = 5 μm (see ref. 14 and 15, for instance, for MIC_0_). For simplicity, the number of trapped peptides *N*_p_ is chosen to be the same for bacteria and host cells: *N*_p_ = 0, 10^7^, 5 × 10^7^. Otherwise, we choose the same parameters used in Fig. 5: the bacterial cell surface area *A*_B_ = 12 μm^2^ (suitable for *E. coli*); the host cell surface area *A*_H_ = 200 μm^2^ ≈ 17 × *A*_B_ (as for human red blood cells); *a*_H_ = 71 Å^2^ and *a*_H_ = 74 Å^2^; *w*_B_ = −16.6 *k*_B_*T* and *w*_H_ = −6.72 *k*_B_*T*;^18^*v*_p_ = 33^3^ Å^3^ and *A*_p_ = 400 Å^2^.^17,18^

**Fig. 6:**
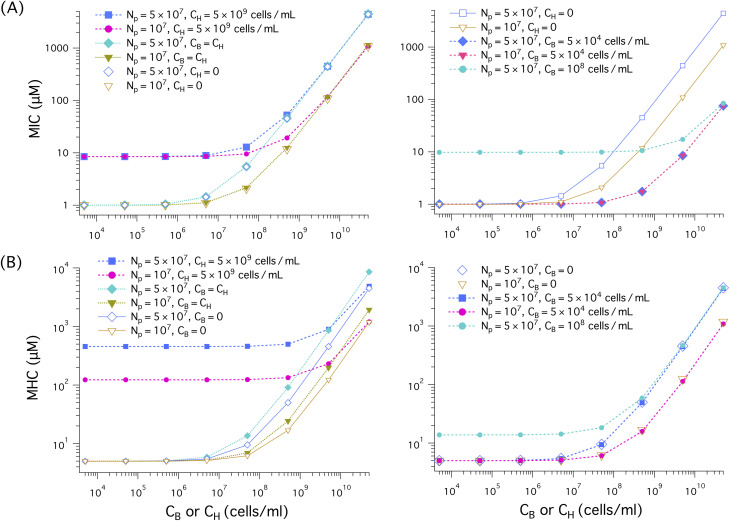
Cell-density dependence of MIC (A) and MHC (B) for the noncompetitive and competitive cases, represented by solid lines with unfilled symbols (or unfilled symbols) and dashed lines with filled symbols, respectively. When *C*_H_ (*C*_B_) is held fixed, the ‘*x*’ axis represents *C*_B_ (*C*_H_); for the case *C*_H_ = *C*_B_, it indicates both *C*_H_ and *C*_B_ (as indicated in the legends of (A), (B), and (C)). We have chosen the parameters as follows: MIC_0_ = 1 μm and MHC_0_ = 5 μm; *w*_B_ = −16.6 *k*_B_*T* and *w*_H_ = −6.72 *k*_B_*T*, as for a melittin-like peptide; the bacterial cell surface area *A*_B_ = 12 μm^2^ (suitable for *E. coli*); the host cell surface area *A*_H_ = 200 μm^2^ ≈ 17 × *A*_B_ (as for human red blood cells); the lipid headgroup area *a*_B_ = 71 Å^2^ and *a*_H_ = 74 Å^2^; *v*_p_ = 33^3^ Å^3^ and *A*_p_ = 400 Å^2^. In all cases shown in (A) and (B), both the MIC and MHC increase with increasing cell density (*C*_H_ or *C*_B_), as expected from eqn 4(a) and (b). (A) (Left) The presence of a large amount of host cells as in whole blood increases the MIC up to ten-fold, as long as *C*_B_ ≲ 5 × 10^8^ cells per mL; for this, compare the curve obtained with *C*_H_ = 5 × 10^9^ cells per mL with the corresponding one obtained with *C*_H_ = 0. Also the MIC increases more rapidly, if *N*_p_ is larger. The MIC remains ≲ 10 μm if *C*_B_ ≲ 5 × 10^7^ cells per mL. If *C*_B_ ≳ 5 × 10^8^ cells per mL, the presence of host cells does not have a significant impact on the MIC; in this case, peptide trapping in bacterial cells is a determining factor. For the same value of *N*_p_, different curves representing different values of *C*_H_ collapse onto each other for sufficiently large *C*_B_: *C*_B_ ≳ 5 × 10^8^ cells per mL. There is no noticeable difference between the two cases: *C*_B_ = *C*_H_ and *C*_H_ = 0 for given *N*_p_. In this case, the main source of inoculum effects is the trapping of peptides in bacterial cells. (A) (right) When *C*_B_ is held fixed at *C*_B_ = 5 × 10^4^ cells per mL, the MIC is insensitive to the value of *N*_p_ used, as if bacterial cells are in the low-cell density limit (*i.e.*, their presence creates a minimal inoculum effect). At the MIC, host cells are below the MHC. As a result, the binding of peptides to the host-cell membrane is responsible for the slow increase of the MIC with *C*_H_. The presence of a large amount of bacterial cells (*C*_B_ = 10^8^ cells per mL) increases the MIC about ten-fold as long as *C*_H_ ≲ 10^8^ cells per mL (the two homogenous MIC curves from the graph in the left are also included for comparison purposes.) (B) (left) In all cases, the MHC increases with increasing cell densities: either *C*_B_ or *C*_H_. When *C*_H_ = 5 × 10^9^ cells per mL, the MHC is large and remains roughly flat as *C*_B_ increases up to *C*_B_ = 10^9^ cells per mL. It is obviously larger for the larger *N*_p_ case (squares or diamonds); it can be two orders of magnitude larger than MHC_0_. Also the MHC is larger for the competitive case *C*_B_ = *C*_H_ compared to the corresponding noncompetitive case *C*_B_ = 0: at the MHC, the bacterial cells are above the MIC and the resulting peptide trapping in the bacterial cells raises the MHC. (B) (right) The presence of a small concentration of bacteria (*i.e.*, *C*_B_ = 5 × 10^4^ cells per mL) does not alter the MHC in any significant way. Also the MHC increases faster with *C*_H_ for larger *N*_p_, as expected from eqn (4b). In the presence of a large amount of bacterial cells (*C*_B_ = 10^8^ cells per mL), the MHC is about three times as large as in the corresponding host-cell only case, as long as *C*_H_ ≲ 10^7^ cells per mL.

Fig. 6 displays the results for the MIC (A) and the MHC (B) for the noncompetitive and competitive cases, represented by solid lines with unfilled symbols or unfilled symbols and dashed lines with filled symbols, respectively. As in Fig. 5, when *C*_H_ (*C*_B_) is held fixed, the ‘*x*’ axis represents *C*_B_ (*C*_H_); for the case *C*_H_ = *C*_B_, it stands for both *C*_H_ and *C*_B_.

In all cases shown in Fig. 6(A) and (B), both MICs and MHCs increase linearly with increasing cell density (*C*_H_ or *C*_B_), similarly to what is shown for model membranes in Fig. 5. This is a natural consequence of the cell-density dependence shown in eqn (4).

As indicated in the graph on the left in Fig. 6(A), the presence of an excess amount of host cells raises the MIC, more so for larger *N*_p_ as long as *C*_B_ ≲ 5 × 10^8^ cells per mL; for this, compare the curve obtained with *C*_H_ = 5 × 10^9^ cells per mL with the one obtained with *C*_H_ = 0. Nevertheless, the MIC remains somewhat smaller than 10 μm if *C*_B_ ≲ 5 × 10^7^ cells per mL. When *C*_B_ ≳ 5 × 10^8^ cells per mL, the presence of host cells does not have a significant impact on the MIC; in this case, peptide trapping in bacterial cells is a determining factor. For the same value of *N*_p_, different curves representing different values of *C*_H_ collapse onto each other for sufficiently large *C*_B_: *C*_B_ ≳ 5 × 10^8^ cells per mL. Also, the MIC obtained with *N*_p_ = 5 × 10^7^ increases more rapidly with cell density than the corresponding one obtained with *N*_p_ = 10^7^ does, as suggested by eqn (4). Finally, for given *N*_p_, there is no noticeable difference between the two cases: *C*_B_ = *C*_H_ (competitive) and *C*_H_ = 0 (bacterial-cell only). The presence of an equal amount of host cells has an insignificant impact on the MIC. At the MIC, the host cells are above the MHC (no trapping in the cells) and their effect on the MIC is expected to be minor (see Section 2.2. for the relative significance of membrane association of peptides *versus* peptide trapping in cells).

As shown in the graph on the right in Fig. 6, when *C*_B_ is held fixed at *C*_B_ = 5 × 10^4^ cells per mL, the MIC is insensitive to the value of *N*_p_ used, as if bacterial cells are in the low-cell density limit (*i.e.*, their presence creates a minimal inoculum effect). At the MIC, the host cells, which are present together with bacterial cells, are below the MHC. As a result, the binding of peptides to the host-cell membrane is responsible for the slow increase of the MIC with *C*_H_. The presence of a large amount of bacterial cells (*C*_B_ = 10^8^ cells per mL) increases the MIC about ten-fold as long as *C*_H_ ≲ 10^8^ cells per mL (the two homogenous MIC curves from the graph in the left are also included for comparison purposes).

Fig. 6(B) shows how the MHC varies as a function of cell density: *C*_B_ or *C*_H_. In all cases, the MHC increases with increasing cell density. When *C*_H_ = 5 × 10^9^ cells per mL, the MHC is large and remains roughly flat as *C*_B_ increases up to *C*_B_ = 10^9^ cells per mL. This is consistent with eqn (4b), which suggests that the MHC is roughly independent of *C*_B_, as long as *C*_H_ is sufficiently larger than *C*_B_. The MHC is obviously larger for the larger *N*_p_ case (squares or diamonds). Finally, the MHC is somewhat larger in the presence of an equal amount of host cells (*C*_B_ = *C*_H_) compared to the host-cell only case *C*_B_ = 0. Peptide trapping in the bacterial cells is responsible for this.

As shown in the graph on the right in Fig. 6, the presence of a small concentration of bacteria (*i.e.*, *C*_B_ = 5 × 10^4^ cells per mL) does not alter the MHC in any significant way. For this, compare open diamonds and filled squares or between open inverted triangles and filled circles. Similarly to the other cases shown on the left in Fig. 6(B), the MHC increases faster with *C*_H_ for larger *N*_p_, as expected from eqn (4b). The presence of a large amount of bacterial cells (*C*_B_ = 10^8^ cells per mL) increases the MHC about three-fold from the corresponding host-cell only case, long as *C*_H_ ≲ 10^7^ cells per mL. For this, compare the dashed curve with filled circles in cyan with open diamonds in blue.

**Fig. 7:**
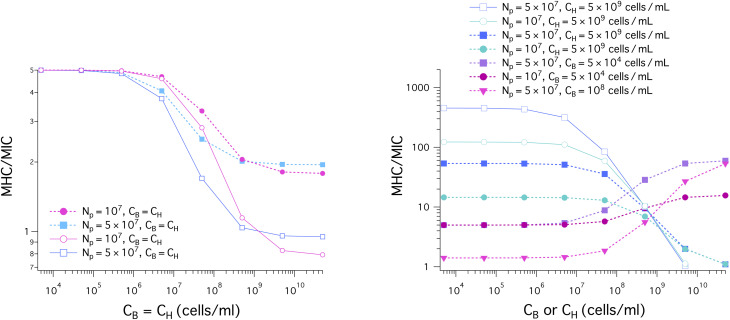
Cell-density dependence of MHC/MIC for the noncompetitive and competitive cases, represented by solid lines with unfilled symbols and dashed lines with filled symbols, respectively. In the graph on the left, *C*_H_ = *C*_B_; in the graph on the right, the ‘*x*’ axis represents *C*_B_ (*C*_H_), when *C*_H_ (*C*_B_) is held fixed. We have chosen the same parameters as in Fig. 6: MIC_0_ = 1 μm and MHC_0_ = 5 μm; *w*_B_ = −16.6 *k*_B_*T* and *w*_H_ = −6.72 *k*_B_*T* as for melittin; the bacterial cell surface area *A*_B_ = 12 μm^2^ (suitable for *E. coli*); the host cell surface area *A*_H_ = 200 μm^2^ ≈ 17 × *A*_B_ (as for human red blood cells); *a*_B_ = 71 Å^2^ and *a*_H_ = 74 Å^2^; *v*_p_ = 33^3^ Å^3^ and *A*_p_ = 400 Å^2^. (left) In all cases shown, *C*_H_ = *C*_B_. The selectivity, MHC/MIC, decreases as the cell density increases. It is larger for the competitive case (filled symbols), more so for larger *C*_H_ = *C*_B_. For *C*_H_ = *C*_B_ ≲ 10^9^ cells per mL, the selectivity is somewhat larger when *N*_p_ is smaller; in this case, peptide trapping works in bacteria's favor by increasing the MIC. (right) The selectivity obtained with *C*_H_ = 5 × 10^9^ cells per mL decreases with increasing *C*_B_, more rapidly for larger *N*_p_. In this case, peptide trapping enhances the selectivity as long as *C*_B_ ≲ 5 × 10^9^ cells per mL (competitive) or *C*_B_ ≲ 5 × 10^8^ cells per mL (noncompetitive) but does not seem to have a noticeable impact outside this range. In contrast, it increases with *C*_H_, more rapidly for larger *N*_p_, when *C*_B_ is held fixed at *C*_B_ = 5 × 10^4^ cells per mL or *C*_B_ = 10^8^ cells per mL. The selectivity is smaller for the latter choice of *C*_B_. With the parameter choices used, the noncompetitive selectivity can be an order of magnitude larger than the corresponding competitive one; depending on how the selectivity is measured, it can be two or three order of magnitude different; for this, compare the blue solid line with open squares with the magenta dashed curve with inverted filled triangles.

Fig. 7 shows the results for MHC/MIC. The dashed lines with filled symbols represent competitive selectivity, whereas the solid lines with unfilled symbols describe noncompetitive selectivity; in the latter case, MHCs and MICs, obtained for host-cell only and bacteria-only solutions, respectively, are combined into MHC/MIC.

In the graph on the left in Fig. 7, we have chosen *C*_H_ = *C*_B_. In all cases shown in the graph, the selectivity, MHC/MIC, decreases from the initial value MHC_0_/MIC_0_ as the cell density increases. It is larger for the competitive case (filled symbols) than for the corresponding noncompetitive case so that the difference between the two cases is more pronounced for larger *C*_H_ = *C*_B_. For *C*_H_ = *C*_B_ ≲ 10^9^ cells per mL, the selectivity is somewhat larger when *N*_p_ is smaller; in this case, peptide trapping works in bacteria's favor by increasing the MIC.

As shown in the graph on the right in Fig. 7, the selectivity obtained with *C*_H_ = 5 × 10^9^ cells per mL decreases with increasing *C*_B_, more rapidly when *N*_p_ is larger. In this case, peptide trapping enhances the selectivity for *C*_B_ ≲ 5 × 10^9^ cells per mL (competitive) or *C*_B_ ≲ 5 × 10^8^ cells per mL (noncompetitive) but does not seem to have a noticeable impact outside this range, as it approaches MHC_0_/MIC_0_. In contrast, it increases with *C*_H_, more so for larger *N*_p_, when *C*_B_ is held fixed at *C*_B_ = 5 × 10^4^ cells per mL or *C*_B_ = 5 × 10^8^ cells per mL. The selectivity is smaller for the latter choice of *C*_B_. The presence of host cells in the competitive case works in favor of the host cells by enhancing the selectivity, more effectively for larger *N*_p_.

The results in Fig. 7 show how the selectivity can be overestimated. With the parameter choices used, the noncompetitive selectivity can be an order of magnitude larger than the corresponding competitive one. Furthermore, depending on how the selectivity is measured, it can be two or three order of magnitude different; for this, compare the solid line with unfilled squares in blue (noncompetitive) and the dashed line with filled squares in purple (competitive).

The picture offered by the graph on the right in Fig. 7 is not only consistent with the earlier observation that the selectivity can be excessively overestimated^11^ (see ref. 16 for a theoretical basis) but also clarifies further how peptide selectivity is influenced by various factors or even the way it is measured: competitive, noncompetitive, the presence of host cells, peptide trapping in dead cells.

### 4.3 Membrane *versus* cell selectivity

There are both similarities and differences between membrane selectivity (Fig. 5) and cell selectivity (Fig. 7) of antimicrobial peptides. In both cases, the membrane-density or cell-density dependence of the selectivity is well manifested. If we set *C*_H_ = *C*_B_, both membrane and cell selectivity decrease with *C*_H_ = *C*_B_. In the presence of 5 × 10^9^ cells per mL of host cells or neutral membranes (mimicking host cell membranes) as in whole blood, the selectivity decreases as *C*_B_ increases. In both cases, the selectivity tends to be overestimated in a noncompetitive environment with reference to the corresponding competitive case; when *A*_H_ = *A*_B_, however, the difference between competitive and noncompetitive selectivity against model membranes appears to be minor, especially when *C*_B_ ≲ 10^9^ cells per mL (Fig. 5). When *C*_B_ is held fixed at *C*_B_ = 5 × 10^4^ cells per mL, the membrane selectivity remains nearly flat as a function of *C*_H_, whereas the cell selectivity increases up to about 10 folds for *N*_p_ = 5 × 10^7^; if *N*_p_ = 0, the selectivity would remain nearly flat (the data not shown).

It is worth noting that the MICs for bacterial membranes in Fig. 5 are much smaller than those for bacterial cells in Fig. 6. In contrast, the MHCs in the two figures are comparable. In the case of *E. coli*, the outer membrane enclosing the cell tends to raise MIC_0_. In addition, peptide trapping in dead cells is also responsible for the differences between membranes and cells. Nevertheless, the qualitative picture offered from membranes (Fig. 5) is generally consistent with the one obtained for cells.

## 5. Discussions and conclusions

We have presented a biophysical model of peptide activity and selectivity by combining a pedagogical approach with a Langmuir-type model. If the former captures the cell-density dependence of peptide activity and selectivity in an intuitively-obvious way, the latter relates peptide binding (or trapping) to an effective binding (or trapping) energy.

Using the model, we have clarified how the presence of host cells and peptide trapping influence peptide selectivity and how competitive selectivity differs from noncompetitive selectivity. If the competitive selectivity represents a mixture of bacteria and host cells, the noncompetitive one is obtained by combining MICs and MHCs for bacterium-only and host-cell-only solutions, respectively. In this work, we chose parameters relevant for the peptide melittin (see refs. ^19–21^ and relevant references therein).

The results based on the model suggest a rather nontrivial dependence of the selectivity on the presence of host cells, cell density, and peptide trapping; these factors or effects can enhance or reduce the selectivity depending on how the density of host cells and that of bacterial cells are chosen. When *C*_B_ = *C*_H_, the selectivity is somewhat smaller for larger *N*_p_, unless *C*_B_ = *C*_H_ is sufficiently large (left graph in Fig. 7). In more general cases (right graph in Fig. 7), however, peptide trapping tends to enhance the selectivity; also the presence of host cells works in favor of the host cells, but it raises the MIC up to about 10-fold (Fig. 6(A)).

When *C*_B_ = *C*_H_, the selectivity decreases from the initial value MHC_0_/MIC_0_, with increasing *C*_B_ = *C*_H_, more rapidly for the noncompetitive case; the selectivity is higher for the competitive case and is not sensitive to the choice of *N*_p_. In the presence of a large amount of host cells (*C*_H_ = 5 × 10^9^ cells per mL), the selectivity decreases with increasing *C*_B_ in both competitive and noncompetitive cases. The noncompetitive selectivity can be one-order of magnitude larger than the corresponding competitive one. When *C*_B_ is held fixed at *C*_B_ = 5 × 10^4^ cells per mL or at *C*_B_ = 10^8^ cells per mL, the competitive selectivity increases with *C*_H_; the selectivity is smaller for the latter choice of *C*_B_. Depending on how cell density is chosen, the selectivity can be overly overestimated – almost by three orders of magnitude.

Our work also clarifies how the cell selectivity of AMPs differs from their membrane selectivity. The selectivity based on model membranes is typically larger than the one measured for cells. In both cases (membranes and cells), noncompetitive selectivity is typically larger than the corresponding competitive one, except for the case *C*_B_ = *C*_H_.

The results in this work suggest that the selectivity reflects not only peptide-membrane parameters but also cell density, peptide trapping, and even the way the selectivity is measured (competitive *vs.* noncompetitive). This is a natural consequence of MICs and MHCs that vary with cell density and *N*_p_. Mapping out possible scenarios of peptide activity and selectivity thus would involve exploring wide ranges of *C*_B_ and *C*_H_, which are not easily realized in experiments.

If the involved peptide-membrane parameters are characterized, our model described by eqn (4) can be used as a predictive model. It enables one to calculate MICs, MHCs, and MHC/MIC, as a function of cell density: *C*_B_ or *C*_H_, the density of bacterial and host cells, respectively.

Alternatively, eqn (3) can be used as a fitting model for analyzing MIC and MHC data obtained in a noncompetitive manner: the ‘*y*’-intercept and the ‘slope’ can be extracted by fitting MIC or MHC data to eqn (3a) or (3b), respectively. This enables one to determine MIC_0_ or MHC_0_. Eqn (12) shows how these quantities are related to peptide's binding energy *w* (*w*_B_ or *w*_H_) and 
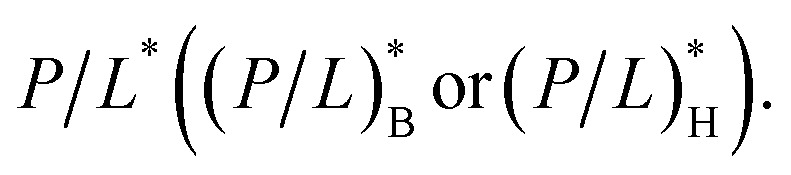
 It is worth noting that *P*/L* has been measured for various model membranes^19–21^ as well as for cells.^10^ Once *P*/L* is known, MIC_0_ and MHC_0_ can be converted into *w*_B_ and *w*_H_, respectively. Conversely, if *w* is known, *P*/L* can be estimated. If all this information is used in the ‘slope,’ the value of *N*_p_ can be extracted.

The information from the homogeneous analysis above can be used in eqn (4), which represents a competitive case. Accordingly, one can quantify peptide selectivity for a biologically relevant setting, which reflects the degree and location of infection. For instance, *C*_B_ ranges from 1 colony-forming unit (CFU mL^−1^) (in blood stream, where *C*_H_ ≈ 5 × 10^9^ cells per mL) to 10^9^ CFU mL^−1^ (in soft tissue or peritonea) (see a recent review^12^ and relevant references therein).

To advance our model and to take fuller advantage of its predictive power, computational and experimental methods can be employed to evaluate further the respective roles of host cells, cell density, and peptide trapping in the selectivity of AMPs (see Fig. 6). Because of their complexity, peptide-cell systems are not so amenable to microscopic computational modeling based on molecular dynamics simulations.^32^ A concerted effort between theoretical modeling, computational approaches, and experiments would be desired. Along the line of what was done in recent studies,^14^ in which a number of key parameters including *N*_p_ were extracted, parameters for multi-species cultures can be mapped out and used in eqn (4) or its variation.

In this work and in a typical experimental setting, the total number of AMPs is treated as a constant. In reality, however, it is influenced by the expression of AMPs by the host^14^ and peptide degradation by protease.^12,22^ Furthermore, earlier studies highlight the stochastic nature of eliminating bacteria with AMPs and its impact on the survivability of a population.^14^ It was shown that below the MIC, two sub-populations emerged: one group that stopped dividing and another group that could grow unharmed and divide. To clarify the roles of these population fluctuations, stochastic modeling of population dynamics can be employed.^33,34^

## Conflicts of interest

There are no conflicts of interest to declare.

## Supplementary Material
